# Identification of Biomarkers and Immune‐Metabolic Regulators in Acute Pancreatitis and Sarcopenia: A Multi‐Modal Transcriptomics Study

**DOI:** 10.1111/jcmm.70769

**Published:** 2025-08-10

**Authors:** Shihang Zhang, Cheng Hu, Xinwei Wang, Zixing Huang, Qing Xia, Lihui Deng

**Affiliations:** ^1^ West China Centre of Excellence for Pancreatitis Institute of Integrated Traditional Chinese and Western Medicine, West China Hospital, Sichuan University Chengdu China; ^2^ Department of Radiology West China Hospital, Sichuan University Chengdu China

**Keywords:** acute pancreatitis, diagnostic biomarker, sarcopenia, transcriptional signature

## Abstract

Studies suggest a clinically significant association between acute pancreatitis and sarcopenia. However, the molecular mechanisms behind this association have not been fully elucidated. Here, we systematically investigated gene expression profiles by differentially expressed gene (DEG) analysis, weighted gene co‐expression network analysis (WGCNA) and functional enrichment analysis, and identified a total of 36 genes as shared genes between acute pancreatitis and sarcopenia. Functional enrichment analysis revealed that these genes were enriched in immune‐inflammatory processes and pathways. Furthermore, we evaluated relevant hub genes in a random forest model and investigated their expression, diagnostic performance and immune cell relationships. Random forest modelling prioritised chloride intracellular channel 5 (*CLIC5*), solute carrier family 38 member 1 (*SLC38A1*) and complement C1q B chain (*C1QB*) as key candidate biomarkers. Immune infiltration analysis linked these genes to dysregulated T cells, monocytes and mast cells in both diseases. Finally, we constructed a regulatory network involving miRNAs, mRNAs and transcription factors to illustrate further the regulations of three genes' transcription in acute pancreatitis and sarcopenia. The diagnostic value of *CLIC5*, *SLC38A1* and *C1QB* was performed by receiver operating characteristic curves and the area under the curve in the datasets, and the validation results confirmed a consistent trend of downregulation of *CLIC5* and *SLC38A1* in AP and sarcopenia. This study revealed for the first time that *CLIC5* and *SLC38A1* were shared biomarkers for AP and sarcopenia. Their association with immune‐metabolic dysregulation highlights their potential as therapeutic targets.

## 1 Introduction

Acute pancreatitis (AP) represents a potential life‐threatening inflammatory disease of the exocrine pancreas caused by intracellular premature activation of trypsinogen, leading to local and systemic inflammation. It is characterised pathologically by localised oedema, inflammatory infiltration and parenchymal necrosis. Clinical manifestations vary from an even disease course, systemic inflammatory response syndrome, to lethal multiple organ failure [1]. Approximately 20% of AP cases progress to severe acute pancreatitis, resulting in a mortality rate of 36%–50% [2].

Sarcopenia is a progressive and systemic skeletal muscle disorder characterised by a significant loss of muscle mass, strength and function [3, 4, 5]. Studies have revealed that sarcopenia increases the risks for unfavourable consequences in diverse diseases, including falls, loss of function, frailty, declined quality of life and increased mortality [6, 7]. An increasing number of studies have shown that sarcopenia is associated with inflammatory digestive disease, of which sarcopenia in pancreatic cancer and pancreatitis has gained much attention. As an acute inflammatory disease of the exocranial pancreas, AP, especially in critically ill patients with organ failure, is associated with inflammation, metabolic disorder, inadequate nutrition and physical inactivity, and can lead to rapid muscle loss, which results in the occurrence of sarcopenia [8]. This acute inflammatory muscle loss is also closely associated with clinical deterioration in AP [9, 10, 11, 12, 13, 14]. Sarcopenia not only exacerbates the condition through a pro‐inflammatory response, but is also significantly associated with a higher risk of death, worse CT imaging severity scores and complications, such as pancreatic necrosis in AP [15, 16, 17]. In addition, sarcopenia may promote the conversion of acute pancreatitis to severe illness, and patients with low muscle mass are more likely to have a poor prognosis for SAP and moderately severe acute pancreatitis [18, 19].

These studies suggest a clinically significant association between AP and sarcopenia. However, the molecular mechanisms linking AP and sarcopenia have not been studied and are yet to be explored. To fill this gap, we analysed differentially expressed genes in patients with AP and sarcopenia by integrating multiple analyses and explored the molecular mechanisms associated with the two diseases through enrichment analysis, immune cell infiltration and miRNA‐mRNA‐TF regulatory networks construction, providing novel insights to improve the therapeutic strategies.

## 2 Methods

### 2.1 Data Collection and Processing

**FIGURE 1:**
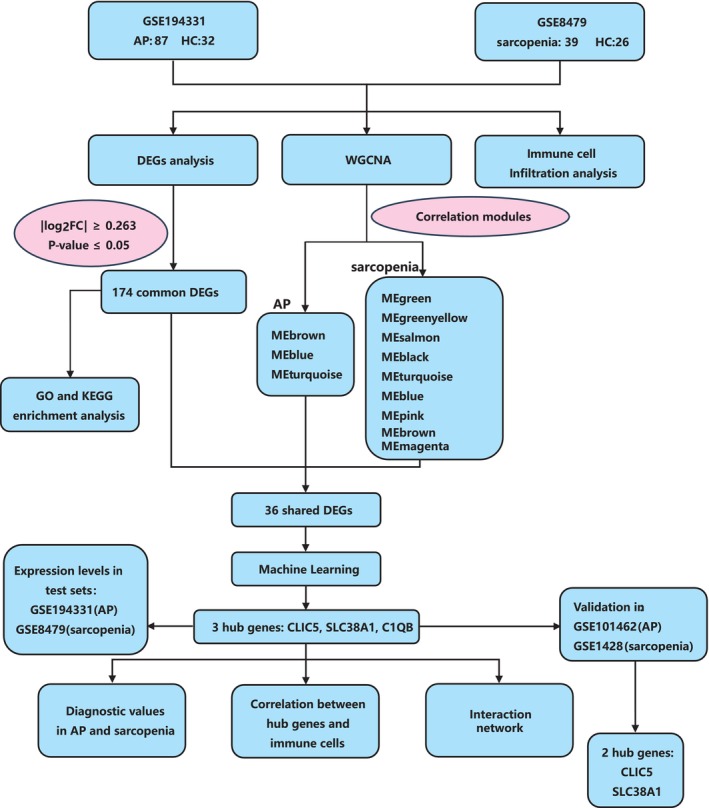
Study flowchart. AP, acute pancreatitis; DEGs, differentially expressed genes; GO, Gene Ontology; HC, healthy control; KEGG, Kyoto Encyclopedia of Genes and Genomes; ME, module eigengene; WGCNA, weighted gene co‐expression network analysis.

The overview of the study flow chart is shown in Figure 1. In this study, we obtained the gene expression profiling data of AP (GSE149331 and GSE101462) and sarcopenia (GSE8479 and GSE1428) from Gene Expression Omnibus (GEO, http://www.ncbi.nlm.nih.gov/geo). GSE149331 and GSE8479 were used as test sets to identify intersecting genes associated with AP and sarcopenia. GSE101462 and GSE1428 were used as validation sets to confirm the hub genes. Every dataset was normalised using normalizeBetweenArrays of the limma package in R during analysis to eliminate technical differences between different chips and make the data more comparable.

### 2.2 Identification of Differentially Expressed Genes (DEGs) and Functional Enrichment Analysis

DEGs in the expression profiles of AP (GSE194331) and sarcopenia (GSE8479) were identified by using the R package ‘limma’, setting the selection criteria as adjusted *p* value ≤ 0.05 and |log_2_ fold change (FC)| ≥ 0.263, corresponding to a fold change ≥ 1.2 [20]. To visualise DEGs, we used the R packages ‘ggplot2’ and ‘pheatmap’ to draft volcano plots and gene clustering heatmaps. We used the ‘Venn’ package to identify the number of genes with consistent expression trends between AP and sarcopenia. Functional enrichment analysis of DEGs was conducted through Gene Ontology (GO) and Kyoto Encyclopedia of Genes and Genomes (KEGG) analyses by using the ‘clusterProfiler’ package in R.

### 2.3 Weighted Gene Co‐Expression Network Analysis (WGCNA)

WGCNA was performed using the R package ‘WGCNA’ to identify the biologically significant co‐expression modules and to investigate the association between gene networks and disease: (1) optimal soft threshold selection for scale‐free network construction. We systematically tested power values from 1 to 10 and 12 to 20 (in increments of 2) using the pickSoftThreshold function. The power value achieving a scale‐free topology fit (signed *R* ^2^) ≥ 0.9 was selected, as determined by plotting –sign (*R* ^2^) versus power values and applying a cut‐off at *R* ^2^ = 0.9; (2) module identification via the dynamic tree‐cutting algorithm. Modules were identified using the blockwiseModules function with parameters: minModuleSize = 50. (3) module merging using the dynamic hybrid method. Similar modules were merged using the dynamic hybrid method with mergeCutHeight = 0.05. (4) trait–module correlation analysis based on Pearson coefficients between module eigengenes and sample characteristics. Key module genes from WGCNA were intersected with DEGs through Venn analysis to identify core genes associated with both AP and sarcopenia.

### 2.4 Feature Selection of Candidate Biomarkers via Random Forest Model

We applied the Random Forest machine learning algorithm to the expression profiles of AP and sarcopenia using the ‘randomForest’ software package to calculate the relative importance score of each gene with the following parameters and procedures: initial models were constructed with ntree = 500 trees. The optimal number of trees was determined by identifying the minimising error using which.min. Genes were ranked by variable importance score, and based on the recent article, those with importance > 1 were selected based on standard biomarker selection practice [21]. The top 15 genes were visualised using ggplot2 dot plots. Candidate genes were identified using the intersection of these genes in the random forest models constructed for AP and sarcopenia.

### 2.5 Immune Cell Infiltration Analysis

We assessed immune cell infiltration in patients with AP and sarcopenia using the R package CIBERSORT, a deconvolution algorithm that estimates the abundance of 22 human immune cell subtypes based on gene expression profiles and further compared the differences between the disease and control groups [22]. The LM22 signature matrix was used as a reference, which contains gene expression signatures for 22 immune cell types. Then differential immune cell infiltration between disease and control groups was compared using the Wilcoxon rank‐sum test, and correlations between hub genes and immune cells were evaluated by correlation analysis.

### 2.6 Construction of miRNA‐mRNA‐Transcription Factor (TF) Interaction Network

By integrating the target regulation of miRNA on mRNA, the regulation of gene expression by TF and their feedback relationships, the miRNA‐mRNA‐TF interaction network can help to identify the key regulatory nodes and pathways in disease progression, and to provide a basis for analysing the pathological mechanisms [21]. To construct the miRNA‐mRNA‐TF regulatory network, we employed the NetworkAnalyst 3.0 platform (https://www.networkanalyst.ca/) for the data collection. After integrating the filtered mRNAs, miRNAs and TFs, the regulatory network was visualised by using Cytoscape for comprehensive analysis.

### 2.7 Diagnostic Evaluation and Expression Analysis of Candidate Biomarkers

We used the ‘pROC’ package to construct receiver operating characteristic (ROC) curves and calculate the area under the curve (AUC) to evaluate the diagnostic accuracy of the filtered genes. An AUC score below 0.7 was considered poor, and a score greater than 0.7 or higher was considered acceptable or better. A multigene diagnostic model was developed using logistic regression analysis, and ROC curves were plotted to evaluate the performance of the multigene diagnostic model. To validate these results, we assessed the expression of the hub genes in the test (GSE149331) and validation dataset (GSE101462) of patients with AP and in the test (GSE8479) and validation dataset (GSE1428) of patients with sarcopenia.

## 3 Results

### 3.1 Identification of Common DEGs in Patients With AP and Sarcopenia

**FIGURE 2:**
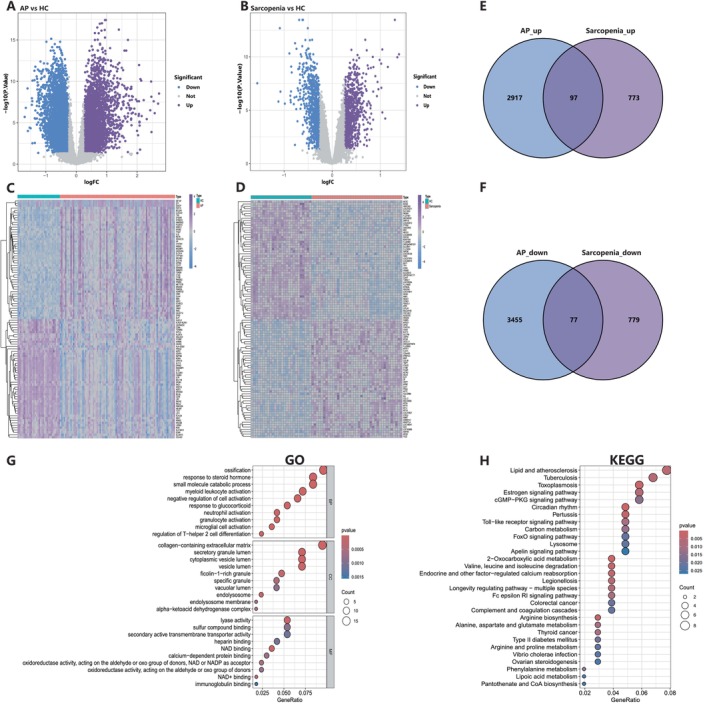
Identification of DEGs in acute pancreatitis (GSE194331) and sarcopenia (GSE8479) patients. (A, B) Volcano plots of all DEGs in GSE5194331 and GSE8479, blue indicates downregulated DEGs, and purple indicates upregulated DEGs. (C, D) A heatmap of all DEGs in GSE194331 and GSE8479. (E, F) Venn diagram identifies co‐upregulated and co‐downregulated DEGs. (G, H) The enrichment analysis results of GO and KEGG pathway. AP, acute pancreatitis; HC, health volunteers; GO, Gene Ontology; KEGG, Kyoto Encyclopedia of Genes and Genomes.

According to the methods established, we identified 6546 DEGs from dataset GSE194331 of patients with AP, including 3014 upregulated and 3532 downregulated genes. These DEGs were visualised using volcano plots and heatmaps (Figure 2A,C). From dataset GSE8479 of patients with sarcopenia, 1726 DEGs, including 870 upregulated and 856 downregulated genes, were identified and visualised in heatmaps and volcano plots (Figure 2B,D). A total of 174 common DEGs with similar expression trends were identified through the Venn diagram intersection, including 97 upregulated and 77 downregulated genes (Figure 2E,F).

By using 174 common DEGs, we further performed GO and KEGG pathway enrichment analyses to explore biological features and pathways. GO analysis revealed that key enriched processes of Biological Processes (BP) included ‘response to steroid hormone’, ‘response to glucocorticoid’ and ‘myeloid leukocyte activation’, highlighting the roles of hormonal regulation and immune responses. Other significant terms included ‘ossification’, ‘small molecule catabolic process’ and ‘regulation of T‐helper 2 cell differentiation’, reflecting metabolic and immune‐related changes. In Cellular Components (CC), the most enriched term was ‘collagen‐containing extracellular matrix’, emphasising extracellular matrix remodelling. Other significant terms, such as ‘secretory granule lumen’, ‘cytoplasmic vesicle lumen’ and ‘endolysosome membrane’, suggested the alterations in vesicular and lysosomal functions. In Molecular Functions (MF), enriched terms included ‘lyase activity’, ‘NAD binding’ and ‘sulfur compound binding’, indicating changes in enzymatic and binding activities. ‘Oxidoreductase activity’ and ‘calcium‐dependent protein binding’ were also significant, highlighting redox and calcium‐mediated processes. The KEGG pathway enrichment analysis revealed that the most significant pathways included ‘Lipid and atherosclerosis’, ‘Tuberculosis’ and ‘Toxoplasmosis’. Immune‐related pathways, such as ‘Toll‐like receptor signaling pathway’ and ‘Pertussis’, were also enriched, highlighting the role of immune responses. Metabolic pathways like ‘Carbon metabolism’, ‘FoxO signaling pathway’ and ‘Valine, leucine, and isoleucine degradation’ were significant, reflecting alterations in metabolic processes. Additionally, signalling pathways, such as ‘Estrogen signaling pathway’ and ‘cGMP‐PKG signaling pathway’, were enriched, suggesting their involvement in disease mechanisms. These results demonstrated a correlation between AP and sarcopenia, and processes related to inflammation, immune responses and lipid metabolism.

### 3.2 Identification of Hub Genes Associated With AP and Sarcopenia Using WGCNA

**FIGURE 3:**
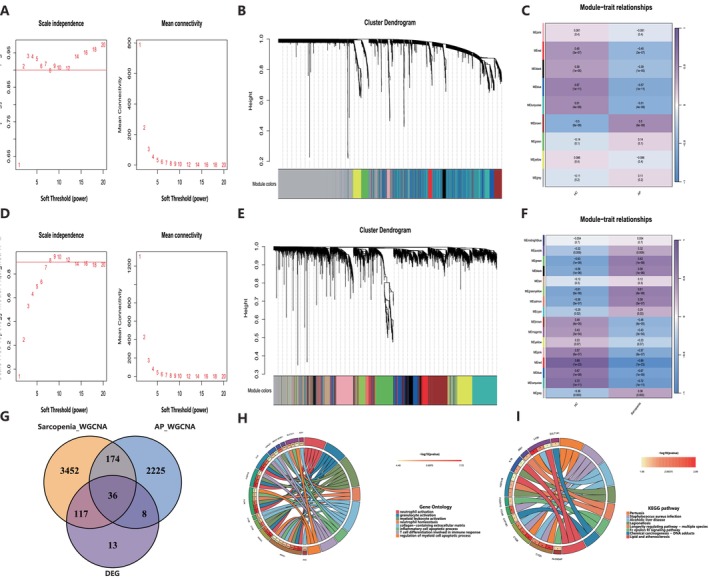
WGCNA analysis of the significant modules in acute pancreatitis and sarcopenia. (A, D) The selection of soft threshold in acute pancreatitis (GSE194331) and sarcopenia (GSE8479). (B, E) Gene cluster dendrogram for acute pancreatitis and sarcopenia by dynamic tree cut algorithm. (C, F) Heatmap of the association between modules and clinical traits in acute pancreatitis and sarcopenia. (G) Venn diagram demonstrates the intersection of common genes obtained by WGCNA and DEGs. (H, I) The chord diagram shows significantly enriched top 8 signalling pathways in GO and KEGG and distribution of DEGs in each pathway.

Previous studies have proved that WGCNA is a powerful approach for finding key co‐expressed gene modules related to specific clinical and biological features of interest [23]. In this study, we performed WGCNA on datasets GSE194331 and GSE8479 to identify key modules associated with AP and sarcopenia. The optimal soft thresholding value *β* = 8 was chosen (Figure 3A,D). According to similarity‐based clustering, we use a dynamic tree cut‐off threshold of 0.05 for module merging. We identified 9 modules in the GSE194331 dataset and 16 modules in the GSE8479 dataset, respectively (Figure 3B,E). Then we calculated the Pearson correlation coefficients between MEs and sample traits. The brown module showed a positive correlation with AP (*r* = 0.5), while the blue (*r* = −0.57) and the turquoise (*r* = −0.51) modules showed negative correlations (Figure 3C). For sarcopenia, the green module (*r* = 0.63), the greenyellow (*r* = 0.61), the salmon (*r* = 0.58) and the black (*r* = 0.56) modules showed high positive correlations, while the red module (*r* = −0.89), the turquoise (*r* = −0.72), the blue (*r* = −0.67) and the pink (*r* = −0.57) modules showed negative correlations (Figure 3F). Finally, we intersected the disease‐relevant module genes identified by WGCNA in AP and sarcopenia with the DEGs acquired from differential expression analysis, finding a total of 36 DEGs between AP and sarcopenia (Figure 3G).

We further analysed these 36 DEGs for GO and KEGG enrichment to explore the shared regulatory pathways. GO analysis revealed that the DEGs were enriched in immune and inflammatory response processes, such as ‘neutrophil activation’, ‘granulocyte activation’ and ‘inflammatory cell apoptosis’. The ‘collagen‐containing extracellular matrix’ was significantly enriched, suggesting tissue damage and repair mechanisms. Processes like ‘T cell differentiation’ and ‘regulation of myeloid cell apoptotic process’ were enriched, indicating the involvement of adaptive immunity and apoptotic regulation (Figure 3H). KEGG pathway analysis of shared DEGs revealed that the DEGs were enriched in immune, metabolic and stress‐related pathways (Figure 3I). For instance, ‘Fc epsilon RI signaling pathway’, ‘Pertussis’ and ‘ *Staphylococcus aureus* infection’ were enriched, highlighting immune activation in both conditions. ‘Alcoholic liver disease’, ‘Chemical carcinogenesis‐DNA adducts’ and ‘Lipid and atherosclerosis’ were identified, suggesting metabolic dysregulation and cellular stress. The ‘Longevity regulating pathway‐multiple species’ was enriched, indicating a role for cellular ageing and stress. In summary, GO and KEGG analyses implicated immune dysregulation, metabolic stress and cellular ageing in the shared pathogenesis of AP and sarcopenia.

### 3.3 Identification of Candidate Biomarkers Using a Random Forest Model

**FIGURE 4:**
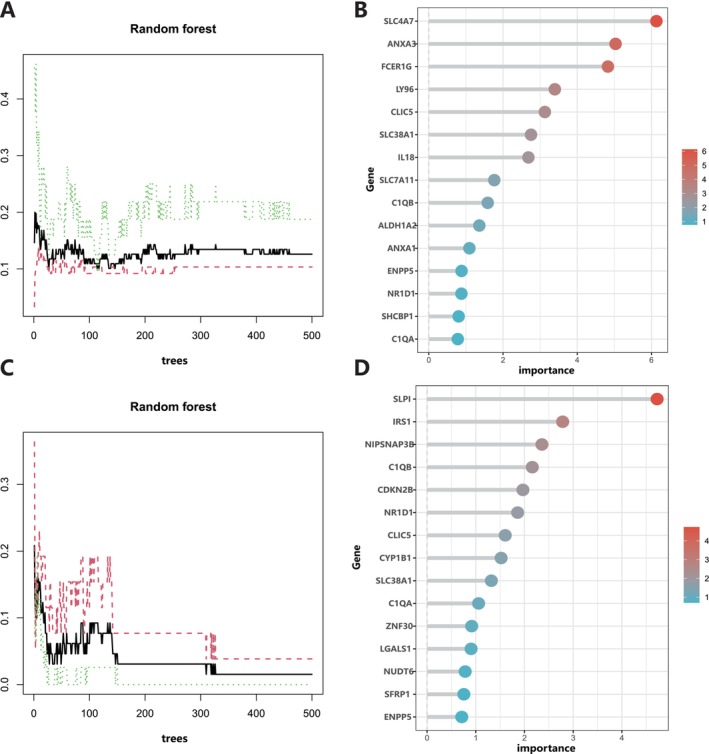
Identification of candidate biomarkers via machine learning. (A, B) Based on the random forest algorithm, the correlation between the total number of trees and the error rate in the acute pancreatitis dataset, as well as the ranking of gene relative importance scores. (C, D) Based on the random forest algorithm, the correlation between the total number of trees and the error rate in the sarcopenia dataset, as well as the ranking of gene relative importance scores.

To further identify potential genes for patients with AP and sarcopenia, we used the random forest machine learning algorithm to analyse these 36 related genes, in which relative relevance scores greater than one were chosen. In the AP dataset, the random forest model identified 11 genes, including solute carrier family 4 member 7 (*SLC4A7*), annexin A3 (*ANXA3*), Fc receptor, IgE, high affinity I, gamma polypeptide (*FCER1G*), lymphocyte antigen 96 (*LY96*), chloride intracellular channel 5 (*CLIC5*), solute carrier family 38 member 1 (*SLC38A1*), interleukin 18 (*IL18*), solute carrier family 7 member 11 (*SLC7A11*), complement C1q B chain (*C1QB*), aldehyde dehydrogenase 1 family member A2 (*ALDH1A2*) and annexin A1 (*ANXA1*) (Figure 4A,B). In the sarcopenia dataset, the random forest model identified 10 genes, including secretory leukocyte peptidase inhibitor (*SLPI*), insulin receptor substrate 1 (*IRS1*), nipsnap homologue 3B (*NIPSNAP3B*), *C1QB*, cyclin dependent kinase inhibitor 2B (*CDKN2B*), nuclear receptor subfamily 1 group D member 1 (*NR1D1*), *CLIC5*, cytochrome P450 family 1 subfamily B member 1 (*CYP1B1*), *SLC38A1*, and complement C1q A chain (*C1QA*) (Figure 4C,D). By using two models, three overlapping genes were identified: *CLIC5*, *SLC38A1* and *C1QB*. They were used as candidate genes for further analysis.

### 3.4 Immune Cell Infiltration and Its Correlation With Key Related Genes

**FIGURE 5:**
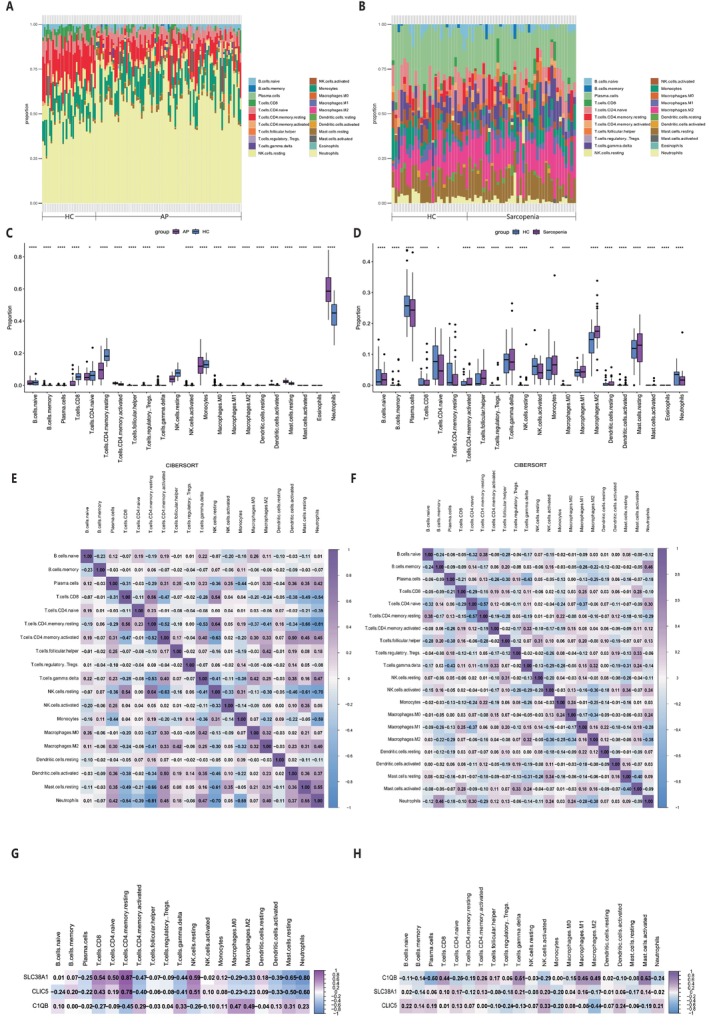
Analysis of immune cell infiltration. (A, B) Stacked bar chart of immune cell infiltration proportions in tissues of patients with acute pancreatitis and sarcopenia. (C, D) Box plot of immune cell proportions between the acute pancreatitis and sarcopenia disease groups and the control group. *p < 0.05; **p < 0.01; ***p < 0.001; ****p < 0.0001. (E, F) Heatmap of correlations between different immune cells in tissues of patients with acute pancreatitis and sarcopenia. The numbers in the squares represent the correlation coefficients between the corresponding immune cells. (G, H) Correlation analysis between infiltrating immune cells and hub genes in tissues of patients with acute pancreatitis and sarcopenia.

As DEGs in immune response‐related pathways were enriched in patients with AP and sarcopenia by GO and KEGG functional enrichment analyses, we assessed the immune cell infiltration status in these two datasets. The results showed that the immune cell profiles of patients with AP and sarcopenia were significantly different from those of healthy people, respectively (Figure 5A,B). Compared with healthy individuals, patients with AP had higher levels of mast cell resting and neutrophils, while the levels were lower in CD8^+^ T cells, naïve CD4^+^ T cells, memory CD4^+^ T‐cell resting, NK cells resting and monocytes (Figure 5C). As for sarcopenia, patients had higher levels of naïve B cells, follicular helper T cells, monocytes, M2 macrophages and mast cells resting. When specialised in plasma cells, patients with sarcopenia had lower levels of naïve CD4^+^ T cells, memory CD4^+^ T cells resting, gamma delta T cells, NK cells activated and neutrophils, compared with healthy individuals (Figure 5D). From the results above, we found that the levels of naïve CD4^+^ T cells and memory CD4^+^ T cells resting were decreased in both AP and sarcopenia, while mast cell resting increased. These results suggested that there might be a common immune dysregulation mechanism between the two diseases in terms of immunity and inflammation. Heat maps demonstrated the interactions among various types of immune cells (Figure 5E,F). Then, we explored the correlations between genes and immune cell components in AP and sarcopenia. *C1QB* was positively correlated with memory CD4^+^ T cell‐activated, gamma delta T cell and M2 macrophages, and was negatively correlated with naïve CD4^+^ T cells, memory CD4^+^ T‐cell resting and NK cells resting. *SLC38A1* was positively correlated with CD8^+^ T cells and naïve CD4^+^ T cells, and was negatively correlated with follicular helper T cells, regulatory Tregs T cells, M2 macrophages, mast cells resting and neutrophils. *CLIC5* was positively correlated with memory B cells, CD8^+^ T cells, naïve CD4^+^ T cells, memory CD4^+^ T cells resting and NK cells activated, and was negatively correlated with follicular helper T cells, regulatory Tregs T cells, gamma delta T cells, M2 macrophages and mast cells resting (Figure 5G,H). These findings indicated that *C1QB*, *SLC38A1* and *CLIC5* might play a key role in the inflammatory responses of immune cells in AP and sarcopenia.

### 3.5 Construction of miRNA‐mRNA‐TF Interaction Network

**FIGURE 6:**
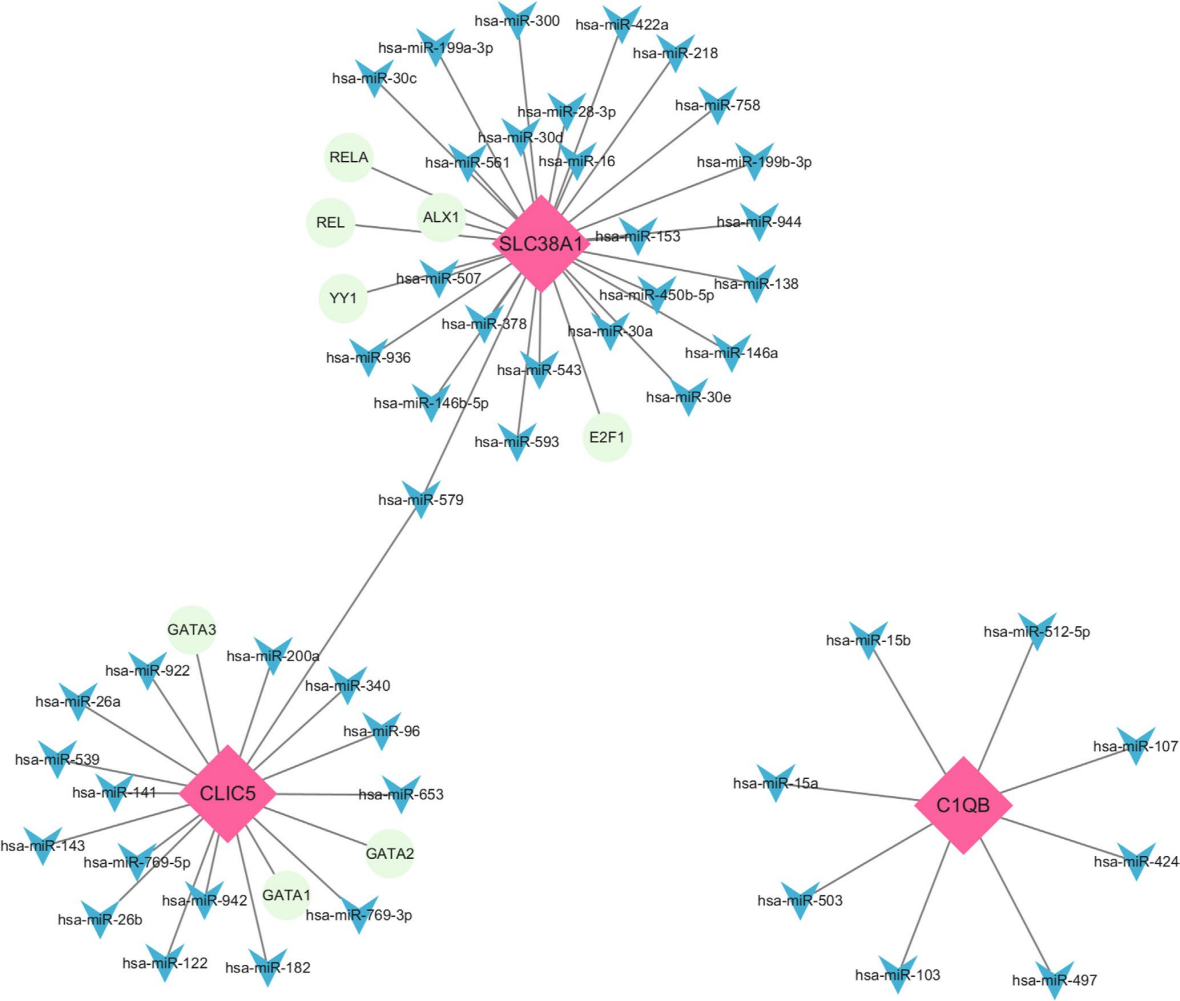
miRNA‐mRNA‐TF regulatory network was constructed based on three immune‐related key genes (*CLIC5*, *SLC38A1* and *C1QB*). Blue arrows represent miRNAs, pink diamonds denote mRNAs, and green circles signify TFs. TF, transcription factors.

Previous studies have shown that miRNA‐mRNA‐TF regulatory networks play an important role in identifying key biomarkers and illustrating disease progression [21, 24, 25]. First, we predicted miRNA‐mRNA interaction pairs, identifying 48 human miRNAs targeting the three key immune‐related genes. Next, we predicted TF‐mRNA interaction pairs and identified eight TFs. Finally, a miRNA‐mRNA‐TF regulatory network was constructed using these three key genes, 48 miRNAs and 8 TFs. In the network, two main transcription factor nodes, *CLIC5* and *SLC38A1*, occupied core positions and interacted with multiple miRNAs and TFs, respectively. *SLC38A1* formed a broad regulatory network with multiple miRNAs (such as miR‐579, miR‐218, miR‐758), TFs (such as RELA, ALX1) and others. *CLIC5* formed a network with multiple miRNAs (such as miR‐579 and miR‐141), TFs (such as GATA1 and GATA2) and others. *C1QB* interacted with miRNAs, such as miR‐503 and miR‐103. These findings indicated their potentially essential roles in AP and sarcopenia (Figure 6).

### 3.6 Diagnostic Evaluation and Expression Analysis of Candidate Biomarkers

**FIGURE 7:**
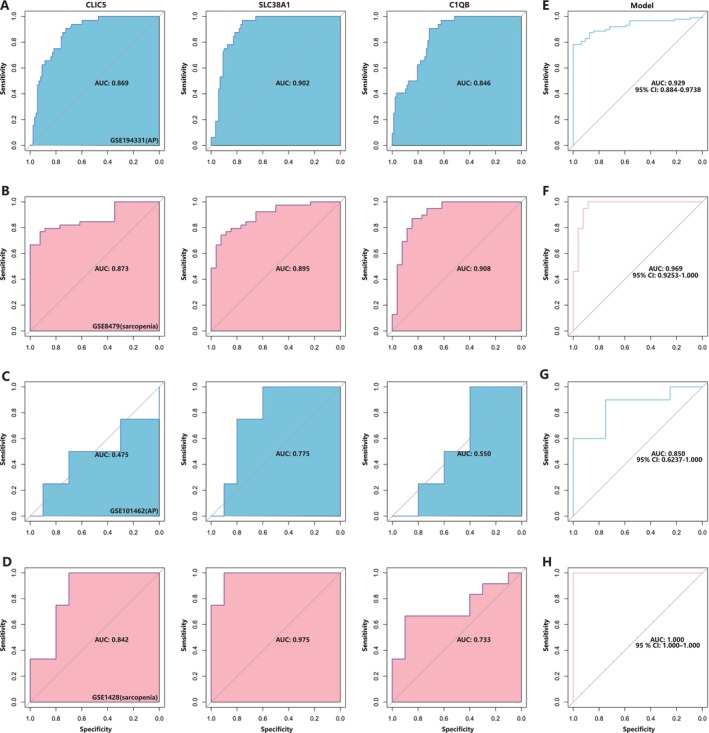
Diagnostic potential of *CLIC5*, *SLC38A1* and *C1QB* in patients with acute pancreatitis and sarcopenia. (A–D) ROC curve of the three shared genes in test and validation datasets for acute pancreatitis and sarcopenia. (E–H) ROC curve of the multi‐marker diagnostic model in test and validation datasets for acute pancreatitis and sarcopenia.

We performed ROC analyses on the different datasets to assess the ability of the three genes to distinguish the disease group from the normal group. The results showed that *CLIC5*, *SLC38A1* and *C1QB* had good abilities to distinguish both AP and sarcopenia from health in the test dataset; however, *CLIC5* and *C1QB* did not show adequate diagnostic abilities in the AP validation dataset (Figure 7A–D). To improve the ability of diagnostic evaluation, we integrated the three hub genes by using logistic regression to create a multi‐marker diagnostic model. The AUC value of this integrated model was 0.929 (95% CI: 0.884–0.9738) in dataset GSE194331, 0.969 (95% CI: 0.9253–1) in dataset GSE8479, 0.850 (95% CI: 0.6237–1) in dataset GSE101462 and 1.000 (95% CI: 1.000–1.000) in dataset GSE1428 (Figure 7E–H), suggesting a good ability of this diagnostic model to distinguish between patients with AP and sarcopenia from healthy individuals.

**FIGURE 8:**
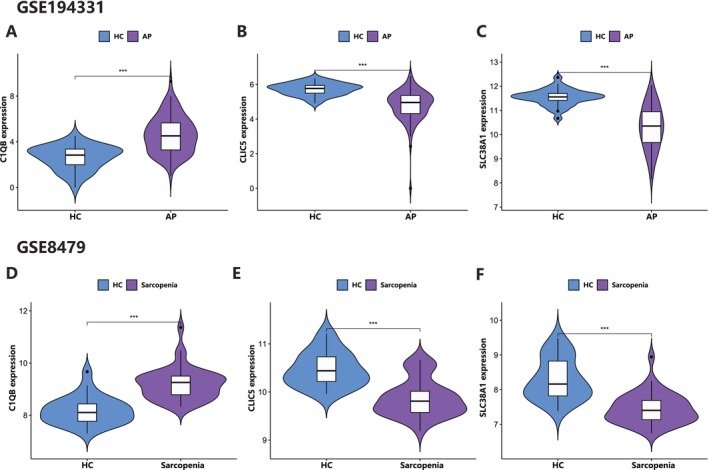
Expression levels of *C1QB*, *CLIC5* and *SLC38A1* in the test datasets of patients with acute pancreatitis and sarcopenia. AP, acute pancreatitis; HC, health volunteers.

**FIGURE 9:**
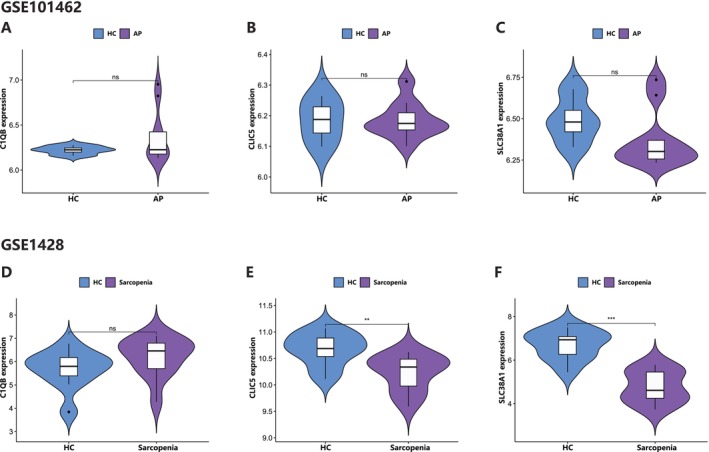
Expression levels of *C1QB*, *CLIC5* and *SLC38A1* in the validation datasets. AP, acute pancreatitis; HC, health volunteers.

To validate the results above, we evaluated the expression of the three hub genes in the test and validation datasets. In both test datasets (GSE149331 and GSE8479), the expression of *CLIC5* and *SLC38A1* was significantly downregulated in AP and sarcopenia, while the expression of *C1QB* was significantly upregulated (Figure 8A–F). As for the validation dataset (GSE101462 and GSE1428), the expression of *CLIC5* and *SLC38A1* in sarcopenia was significantly downgraded, but there was no difference in the expression of *C1QB*. The expression of the three genes was not significantly different in the AP validation dataset, but the decreasing trend of *CLIC5* and *SLC38A1* was similar to the test set (Figure 9A–F). The possible reason for this result may be due to the small sample size of the validation dataset in acute pancreatitis, suggesting that larger samples may be needed in the future. As a result, we think *CLIC5* and *SLC38A1* are the hub genes in these two diseases.

## 4 Discussion

Sarcopenia may occur as age‐related sarcopenia, or as secondary sarcopenia due to non‐ageing factors, such as severe or long‐term inflammation. It is increasingly recognised that sarcopenia may also be caused by acute inflammatory diseases, including AP. However, the common molecular mechanisms of AP and sarcopenia have not been elucidated. To the best of our knowledge, this is the first study that systematically dissected the common molecular mechanisms linking these conditions. The results of this study identified *CLIC5*, *SLC38A1* as common hub genes through integrated multi‐omics analysis, and revealed that immune‐metabolic dysregulation played the central role linking the two diseases. Mechanistic linkage of AP and sarcopenia in our study provides new insights into their common pathogenesis in the future.

In this study, multi‐modal transcriptomics analysis was utilised. Compared with traditional single‐omics analyses, we combined DEGs, WGCNA and machine learning, which improved the accuracy of identifying core genes. By intersecting DEGs with co‐expression modules, we identified 36 core genes. Then, we used random forest modelling to further confirm *SLC38A1* and *C1QB* as biomarkers. Their reliability was also determined by ROC analysis and expression analysis. In addition, we linked transcriptomics to immunisation through immune infiltration analysis and the construction of miRNA‐mRNA‐TF interaction networks. In this way, we not only identified the biomarkers but also revealed the association between immune‐metabolic dysregulation and these diseases.

Although the pancreas and muscle are different tissues, recent research has demonstrated the organ dialogue between the pancreas and muscle. Inflammation of AP can induce muscle loss, and muscle loss can exacerbate inflammation through endocrine pathways, creating a vicious cycle [26]. In sepsis, cytokines released by inflammation reach the muscles via the bloodstream and promote protein degradation in skeletal muscle [27]. Similar to sepsis, we think after AP occurs, inflammatory factors can act on muscles through the bloodstream, triggering protein degradation through related signalling pathways, ultimately leading to muscle loss.

*CLIC5* is a member of the chloride intracellular channel protein family, playing an essential role in regulating the proliferation and differentiation of myoblasts [28, 29]. By binding to the extracellular matrix protein BGN, *CLIC5* was shown to activate the classical Wnt/β‐catenin signalling pathway and regulate skeletal muscle regeneration, while *CLIC5* deficiency in skeletal muscles led to impaired muscle regeneration due to a substantial decline in muscle satellite cell function [29]. In sepsis, *CLIC5* was increased in sepsis‐induced myocardial injury and might play a role in regulating iron metabolism [30]. The changes in *CLIC5* expression in HBEGF^+^ fibroblasts positively correlated with disease status and treatment response in rheumatoid arthritis [31]. The role of *CLIC5* expression has been shown to correlate with the prognosis of cancer, including lung adenocarcinoma, ovarian cancer, pancreatic ductal adenocarcinoma, etc. [32, 33, 34, 35]. The study on *CLIC5* has not been reported in AP or sarcopenia. We found in this study that *CLIC5* was downregulated in the database of patients with AP and sarcopenia. We hypothesise that severe systemic inflammatory response, cytokine storm and metabolic disorders triggered by AP may inhibit *CLIC5* expression and interfere with the proliferation of extracellular matrix through pro‐inflammatory factors, which in turn exacerbate muscle protein degradation and regeneration disorders, and ultimately promote the progression of sarcopenia.

*SLC38A1*, a member of the SLC38 family, is primarily involved in the transport of amino acids, especially glutamine uptake [36]. Glutamine enters cells via SLC38AI transporters to contribute its γ(amide) nitrogen to the synthesis of ribonucleic acid and hexosamine, thereby maintaining the balance of redox reactions and preventing oxidative stress in cells by producing glutathione [37, 38, 39, 40]. Glutamine requirements increase significantly after sepsis, surgery and trauma, and glutamine supplementation has been shown to improve the prognosis by reducing mortality and complications of AP [41, 42]. AP is often accompanied by a systemic inflammatory response and metabolic disturbances, which may lead to metabolic abnormalities and atrophy of muscle tissue, thereby increasing the risk of sarcopenia. We hypothesise that downregulation of *SLC38A1* may play a key role in this process. As a glutamine transporter protein, the downregulation of *SLC38A1* limits intracellular glutamine uptake, which in turn affects amino acid metabolism and protein synthesis, causing the above changes in AP. In sarcopenia, the downregulation of *SLC38A1* leads to inadequate amino acid supply to muscle cells, inhibiting protein synthesis and causing metabolic disorders, which may exacerbate pancreatic cell damage and inflammation after the onset of acute pancreatitis. Therefore, further experiments are needed in the future to verify our speculations.

Our study suggests the correlations of *CLIC5* and *SLC38A1* with AP and sarcopenia; nevertheless, we cannot deny that there are several limitations. First, we did not address whether sarcopenia precedes AP or vice versa. We will propose a future cohort study to solve this problem. Secondly, the lack of clinical data in public datasets prevented us from analysing demographic baselines. This limited our ability to assess potential confounding factors. Future validation should use prospectively collected cohorts and systematic control matching. Thirdly, due to the currently limited research in this field, the sample size of the acquired dataset was relatively small. Finally, although we validated the three shared genes in the database, we did not show the gene expression in the experiments and did not determine whether these genes directly mediate muscle–pancreas interactions or simply reflect systemic immune–metabolic dysregulation through experiments. As we know, *CLIC5* and *SLC38A1* are membrane‐associated conformations and are in low levels in the plasma samples. We can hardly obtain muscle specimens from patients with AP to determine these gene expressions. Currently, a reliable animal model of AP with sarcopenia is still lacking. We intend to investigate the role of these genes in the experiments in the future.

## 5 Conclusion

In conclusion, our study showed *CLIC5* and *SLC38A1* as shared genes of AP and sarcopenia in immune dysregulation and metabolic stress. The common biological processes and potential mechanisms of the two diseases involve inflammatory responses and metabolic disorders.

## Author Contributions

**Shihang Zhang:** data curation (equal), formal analysis (equal), software (equal), writing – original draft (equal). **Cheng Hu:** data curation (equal), writing – review and editing (equal). **Xinwei Wang:** data curation (equal), formal analysis (equal). **Zixing Huang:** conceptualization (equal), methodology (equal). **Qing Xia:** conceptualization (equal), supervision (equal). **Lihui Deng:** conceptualization (lead), funding acquisition (lead), writing – review and editing (lead).

## Conflicts of Interest

The authors declare no conflicts of interest.

## Data Availability

This study analysed publicly accessible datasets, including GSE194331, GSE8479, GSE101462 and GSE1428 datasets from Gene Expression Omnibus (GEO, https://www.ncbi.nlm.nih.gov/geo/query).
